# Defining Dysbiosis in Patients with Urolithiasis

**DOI:** 10.1038/s41598-019-41977-6

**Published:** 2019-04-01

**Authors:** Anna Zampini, Andrew H. Nguyen, Emily Rose, Manoj Monga, Aaron W. Miller

**Affiliations:** 1 Glickman Urological and Kidney Institute, Cleveland Clinic, Cleveland, OH USA; 2 Lerner College of Medicine, Cleveland Clinic, Cleveland, OH USA; 3 Department of Inflammation and Immunity, Lerner Research Institute, Cleveland Clinic, Cleveland, OH USA

## Abstract

The prevalence of urinary stone disease (USD) is rapidly rising. However, the factors driving this increase are unknown. Recent microbiome studies suggest that dysbiosis may in part contribute to the increasing prevalence. The objective of the current study was to determine the nature and location of dysbiosis associated with USD. We conducted microbiome analysis from the gastrointestinal and urinary tracts, along with a metabolomic analysis of the urinary metabolome, from subjects with an active episode of USD or no history of the disease. Higher rates of antibiotic use among USD patients along with integrated microbiome and metabolomic results support the hypothesis that USD is associated with an antibiotic-driven shift in the microbiome from one that protects against USD to one that promotes the disease. Specifically, our study implicates urinary tract *Lactobacillus* and *Enterobacteriaceae* in protective and pathogenic roles for USD, respectively, which conventional, culture-based methods of bacterial analysis from urine and kidney stones would not necessarily detect. Results suggest that antibiotics produce a long-term shift in the microbiome that may increase the risk for USD, with the urinary tract microbiome holding more relevance for USD than the gut microbiome.

## Introduction

The prevalence of urinary stone disease (USD) is approximately 8.8%, representing a four-fold increase from fifty years ago^1^. Similar increases in prevalence exist for co-morbidities of USD such as metabolic syndrome, cardiovascular disease, diabetes, and asthma^2–5^. These concurrent trends are indicative of systemic changes to public health^6^. Multiple factors have been associated with USD, including socioeconomic status, host genetics, diet, pharmaceuticals, antibiotics, and metabolism^7,8^. The microbiome is strongly associated with all of these factors and is thus implicated as an important mediator of USD^9–15^.

Dysbiosis, the contribution of the microbiome to disease processes^16^, can come in one of three different forms. First, a shift in the microbiome can lead to the emergence of bacteria and functions that cause disease, herein referred to as gain of function dysbiosis. Gain of function dysbiosis results from the overgrowth of pathogens that lead to diseases such as cholera, strep throat, or *E. coli* infection. Second, a shift in the microbiome can lead to the loss of bacteria and functions that protect health, herein referred to as loss of function dysbiosis. Loss of function dysbiosis is inherently more difficult to attribute to a disease process as it is by definition, the absence of specific bacteria from a complex microbiome that causes a disease rather than their presence. Regardless, loss of function dysbiosis is increasingly being recognized as an important contributor to many diseases including inflammatory bowel disease (IBD), obesity, cardiovascular disease, asthma, and others^17–27^. Finally, a combination of loss and gain of function dysbiosis may contribute to or be required for some disease processes. Such is the case with recurrent *Clostridium difficile* infection, in which repeated antibiotic use leads to the depletion of the commensal microbiota, which allows for the proliferation of pathogenic *C. difficile*^28^. To determine if a particular disease or disease process is associated with loss or gain of function dysbiosis is straightforward^29,30^. If antibiotics can alleviate disease symptoms or microbial transplants can cause disease symptoms, then gain of function dysbiosis contributes to the disease process. If antibiotics can cause disease symptoms or microbial transplants can alleviate disease symptoms, loss of function dysbiosis contributes to the disease.

Bacteria both in the urinary and gastrointestinal tracts have been linked to USD through both gain and loss of function mechanisms. Urinary tract infections (UTIs) are caused by diverse bacteria primarily from the *Enterobacteriaceae* family^8,31^. Some UTI-associated bacteria produce urease, which breaks down urea, increases pH, and can lead to struvite stone formation^32–36^. Thus, struvite stone formation is a clear case of gain of function dysbiosis and comprises approximately 15% of all cases of USD^1,37^. In contrast to disease-causing *Enterobacteriaceae*, bacteria from the *Lactobacillus* genus are frequently found in the urinary tract of healthy individuals and have been shown to protect against UTI’s in clinical trials^38,39^.

In addition to urinary tract bacteria, bacteria of the gut are thought to protect against the most common stone type, calcium oxalate stones. Oxalate, which is both commonly consumed in the diet and produced endogenously in the liver, is a central component of approximately 80% of kidney stones^40–43^. Despite the dietary exposure and toxicity associated with excess oxalate, humans do not produce enzymes capable of degrading oxalate and instead rely in part on diverse oxalate-degrading bacteria in the gut^44–47^. The bacterium *Oxalobacter formigenes* uses oxalate as a carbon and energy source for growth, is negatively associated with both USD and urinary oxalate, is sensitive to common antibiotics, and can reduce urinary oxalate when administered orally as a probiotic^48^, all of which implicates loss of function dysbiosis associated with this species and calcium oxalate stone formation. However, colonization by *O. formigenes* ranges from 11–100% in individuals with no history of USD and 0–100% in individuals with USD, with only 55% of studies reporting a significant negative association between *O. formigenes* and urinary oxalate excretion^48^. Therefore, colonization by *O. formigenes* alone is neither necessary nor sufficient to prevent USD or reduce urinary oxalate excretion, which is a risk factor for calcium oxalate stone formation^48,49^. Thus, if loss of function dysbiosis contributes to calcium oxalate stone formation, bacteria other than oxalate-degrading bacteria must also be involved.

Recent studies have shown that oxalate metabolism is driven by a diverse oxalate-degrading microbial network, which can be transferred across mammalian species to significantly and persistently decrease urinary oxalate excretion, more effectively than oxalate-degrading bacteria alone^46,50–53^. Additionally, it has been shown that the gut microbiota in individuals with no history of USD differs from that of USD patients and specifically that USD patients harbor fewer oxalate-degrading genes even when there is no difference in colonization by *O. formigenes*^47,54–56^. Finally, the long-term effects of multiple classes of oral antibiotics is directly associated with USD, with stronger associations for exposure at younger ages^57^. Collectively, these results all point towards antibiotic-driven, loss of function dysbiosis as an under-lying risk factor for USD. However, it is unclear if the microbiota of the gut or urinary tract is more important for stone formation, if natural variation in gut microbiota composition drives the results of the published microbiome studies, or how the microbiota contributes to the urinary metabolome in a manner that facilitates or inhibits stone formation.

While USD encompasses diverse pathologies, the increasing prevalence of USD and the association between USD and oral antibiotic use, suggest that common, dysbiosis-driven factors underlie the diverse pathologies of USD at some level. The objective of the current study was to take a multi-specimen, multi-omic approach to specifically determine (1) if calcium-based and uric acid kidney stones are significantly associated with microbial dysbiosis; (2) the site of microbial activity that is most important for USD; and (3) factors that impact the microbiome in a way that facilitates the onset of USD. The goal of the work is to provide a solid foundation to translate results obtained from microbiome studies associated with USD into effective, persistent, and personalized bacteriotherapies to prevent USD.

## Results

### Clinical cohort of participants

A total of 67 individuals were recruited for the current study, with 43 subjects that had no history of USD, and 24 subjects with an active episode of USD. The USD patients had stones composed of calcium oxalate, calcium phosphate, uric acid, or a mixture of components. We did not recruit patients with a history of struvite stones since these stones are known to be derived from pathogenic *Enterobacteriaceae* bacteria. Consistent with previous reports, the healthy and USD cohorts differed significantly by age, diabetes, 12-month antibiotic use, and family history of USD (Table 1) and is thus an adequate representation of the USD population^57–60^.

**Table 1:** Patient metadata.

Metric	Healthy	USD	P-value	Statistic
No. enrolled	43	24	NA	NA
%Antibiotics used in past 12 months	39.53%	75%	**0.01**	Relative risk, Fisher Exact Test
Age	35.47 +/− 1.73	51.54 +/− 2.53	<**0.001**	Student’s t-test
%Prior USD	0%	75%	<**0.001**	Relative risk, Fisher Exact Test
%Diabetic	2.33%	25%	**0.007**	Relative risk, Fisher Exact Test
%CaOx		43%		
%CaOx + CaPhos		22%		
%CaOx + Uric acid		4%		
%CaPhos		13%		
%Uric acid		17%		
%Family History of USD	26%	41%	**0.016**	Relative risk, Fisher Exact Test
%Antibiotics used in past 30 days	0%	4.17%	0.35	Relative risk, Fisher Exact Test
Height (cm)	171.54 +/− 1.53	171.66 +/− 1.7	0.5	Student’s t-test
Weight (kg)	76 +/− 3.26	81 +/− 6.55	0.59	Student’s t-test
%Female	60%	58%	0.785	Relative risk, Fisher Exact Test
%Gastrointestinal Illness	6.98%	8.33%	1	Relative risk, Fisher Exact Test

P-values for significantly different metrics are bolded. *Values expressed as mean +/− standard error.

### 16S rRNA sequencing and untargeted metabolomics

A total of 199 DNA samples from stool, urine, kidney stones, and cultures generated from urine and kidney stones, were subjected to high-throughput sequencing of the V4 region of the 16S rRNA gene. Sequencing resulted in 12,020,020 high quality sequences, used for downstream analyses. We employed a moderate abundance-based operational taxonomic unit (OTU) filtering strategy that balances removing spurious OTUs with maintaining rare OTUs, as done previously^46,48,50–53,61^. With this strategy, a total of 7,376 (1432 +/− 65 per sample), 3,308 (452 +/− 24 per sample), and 473 (341 +/− 74 per sample) unique operational taxonomic units (OTUs) were defined in the stool, urine, and kidney stone samples, respectively, when DNA was extracted directly from samples. From urine and stone cultures, 2,068 (393 +/− 37 per sample) and 635 (137 +/− 23 per sample) OTUs were defined. All samples were represented by a high abundance of sequence reads (>3,000 for urine, stones, or cultures; >10,000 for stool). Taxonomic assignment at the phylum level ranged from 96% (stones) to >99% (stool; Supplementary Fig. S1), while genus level assignment ranged from 68% (stones) to 86% (urine).

For untargeted urinary metabolomics, 31 samples from healthy individuals and 18 samples from USD patients were analyzed. Analysis resulted in 13,348 high quality and unique spectral features. Of these, 2,110 were assigned putative identification, either with mass spectrometry alone or with tandem mass spectrometry.

### Analysis of the microbiome by specimen-type, technique, and USD status

**Figure 1:**
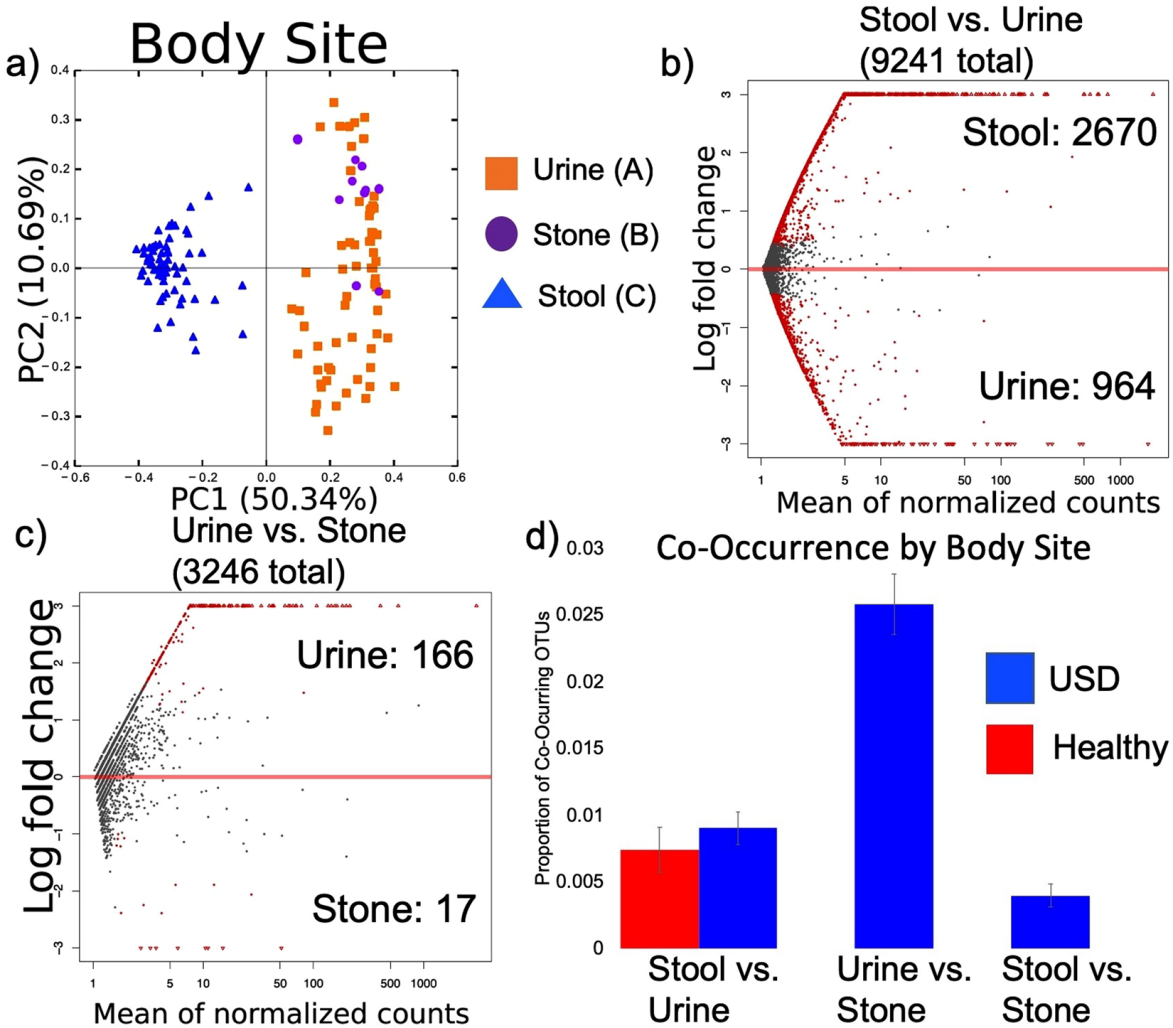
Microbiome analysis by specimen type. (**a**) PCoA plots based on a weighted UniFrac analysis by specimen type, with each principal component listed with % of the dissimilarity explained by the coordinate. Statistical significance was determined by an Adonis with 999 permutations. Letters denote differences with p < 0.05. (**b**,**c**) The differential abundance of OTUs by specimen type as assessed by a negative binomial Wald test. Red dots indicate significantly different OTUs (FDR < 0.05), gray dots indicate non-significant OTUs. Listed are the total number of OTUs defined within the group, along with the number of OTUs enriched in each specimen type. (**d**) The average proportion of OTUs found in both stool and urine, or urine and stone by USD-status. There were no significant differences by group.

The composition of the microbiota from the three specimen-types (urine, stool, and kidney stone) were unique as assessed by beta-diversity (Fig. 1a, Table 2). There was an average of ~0.8% co-occurrence of OTUs between the urine and stool with 39% of the OTUs exhibiting significant differential abundance (Fig. 1b,d). This compares to an average of 2.5% of OTUs co-occurring in both urine and stone with only 6% of the OTUs exhibiting significant differential abundance (Fig. 1c,d).

**Table 2:** List of significant pairwise comparisons of weighted UniFrac beta-diversity.

Comparison	Adonis p-value
Stool vs. Urine	0.008
Stool vs. Stone	0.008
Urine vs. Stone	0.008
Urine by 12 m Antibiotic use	0.022
Urine by Technique	0.008
Stone by Technique	0.048
Urine by USD-status	0.009
Urine by Family history of USD	0.042
Urine by Sex	0.008

Stool/Urine/Stone = Specimen that the microbiome data originated from; USD-status = Healthy vs. USD; Technique = Molecular vs. Culture. P-values were corrected for multiple comparisons using a Holm’s correction.

**Figure 2:**
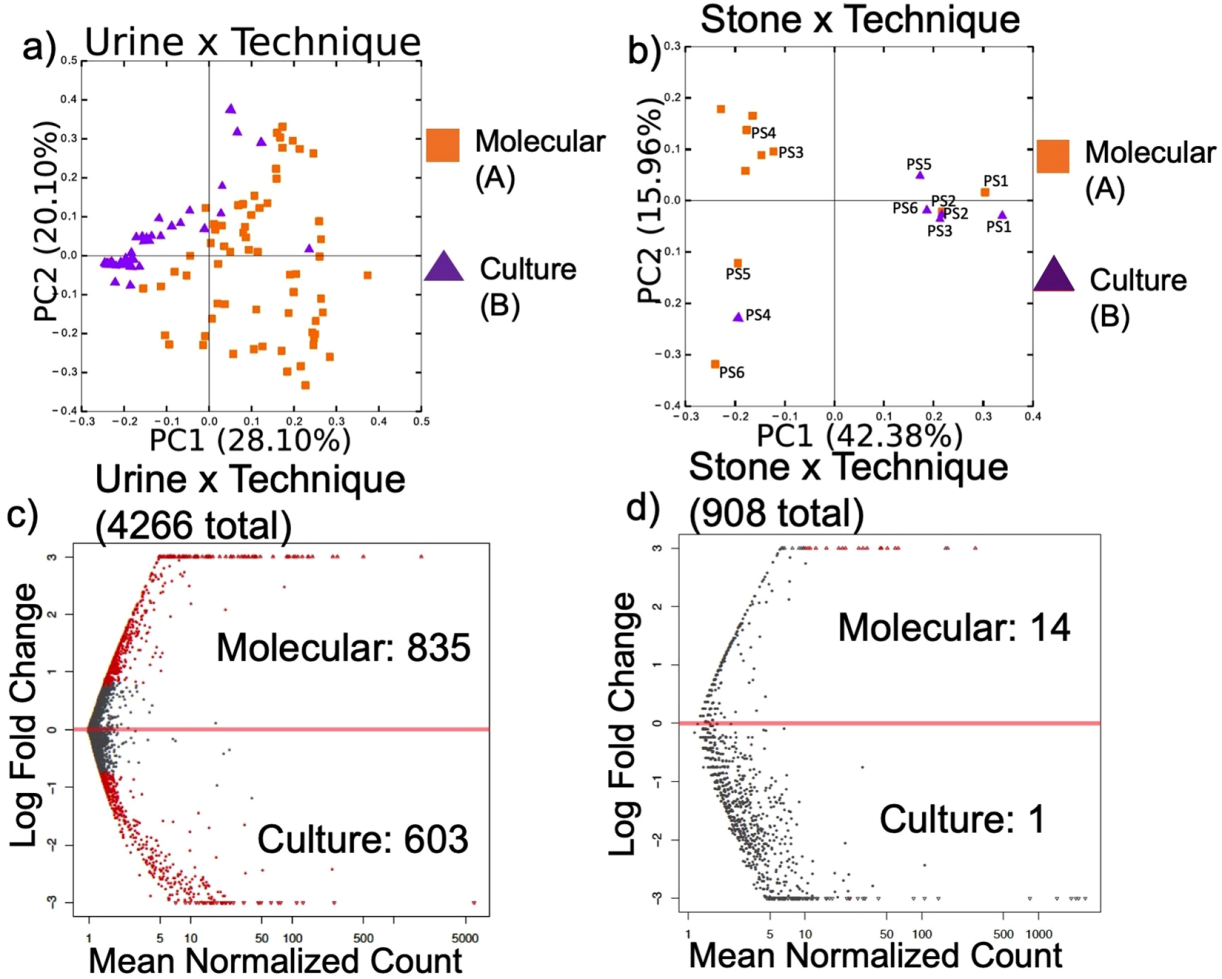
Microbiome analysis by technique. (**a**,**b**) PCoA plots based on a weighted UniFrac analysis by technique for bacterial analysis, with each principal component listed with % of the dissimilarity explained by the coordinate. Statistical significance was determined by an Adonis with 999 permutations. Letters denote differences with p < 0.05. PS = “Paired Sample” and indicates the paired stone samples (molecular vs. culture). (**c**,**d**) The differential abundance of OTUs by technique as assessed by a negative binomial Wald test. Red dots indicate significantly different OTUs (FDR < 0.05), gray dots indicate non-significant OTUs. Listed are the total number of OTUs defined within the group, along with the number of OTUs enriched in each specimen type by group.

To examine the effect of culturing on the bacteria detected by sequencing, we compared 16S rRNA microbial inventories generated from urine and stone samples where DNA had been extracted directly from samples, or after they had been cultured on blood agar (Fig. 2). Bacteria were successfully cultured from only one urine sample and one stone sample on MacConkey’s agar, which we did not include in our data analysis. In blood agar, bacteria were successfully cultured from 30 out of the 43 urine samples of healthy subjects and 19 out of the 24 USD subjects. Additionally, bacteria were successfully cultured in seven of the 10 stone samples. Species detected from urine and stones were dependent on whether bacteria were cultured prior to DNA extraction, with a greater diversity of OTUs detected when DNA was directly extracted from samples for urine but not stone samples (Fig. 2, Supplementary Fig. S1).

**Figure 3:**
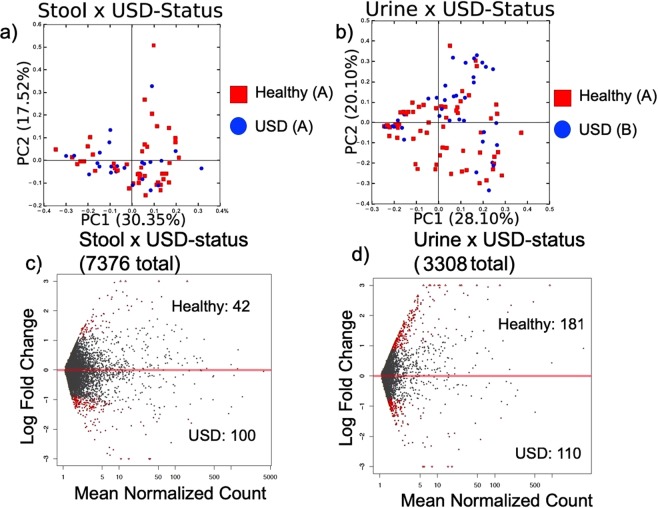
Microbiome analysis by USD-status. (**a**,**b**) PCoA plots based on a weighted UniFrac analysis by USD-status, with each principal component listed with % of the dissimilarity explained by the coordinate. Statistical significance was determined by an ANOSIM with 999 permutations. Letters denote differences with p < 0.05. (**c**,**d**) The differential abundance of OTUs by USD status as assessed by a negative binomial Wald test. Red dots indicate significantly different OTUs (FDR < 0.05), gray dots indicate non-significant OTUs. Listed are the total number of OTUs defined within the group, along with the number of OTUs enriched in each specimen type by group.

For the stool microbiome, there was no difference in beta-diversity of the microbiota between the healthy and USD cohorts (Fig. 3a). However, there was a USD-dependent difference in the composition of the urinary microbiome (Fig. 3b). Furthermore, differential OTU abundance analysis revealed that only 1.9% of the OTUs were significantly different in the stool microbiome between the healthy and USD cohorts with 2.4 fold more OTUs enriched in the USD cohort compared to healthy cohort (Fig. 3c, Supplementary Table S1). For the urinary microbiome, 8.8% of the OTUs were differentially abundant with 1.6 fold more OTUs enriched in the healthy cohort compared to the USD cohort (Fig. 3d, Supplementary Table S1). The taxa that differentiated the healthy cohort from the USD cohort most were the *Lachnospiraceae* in the stool of the USD cohort, *Lactobacillus* in the urine of the healthy cohort, and the *Enterobacteriaceae* in the urine of the USD cohort (Supplementary Table S1).

The composition of the urinary tract microbiome also differed by 12-month antibiotic use, sex, and family history of USD (Table 2). However, urinary tract microbiota composition did not differ significantly by age, diabetic-status, diet, 30-day antibiotic use, height, weight, whether the patient had gout or hypertension (data not shown).

**Figure 4:**
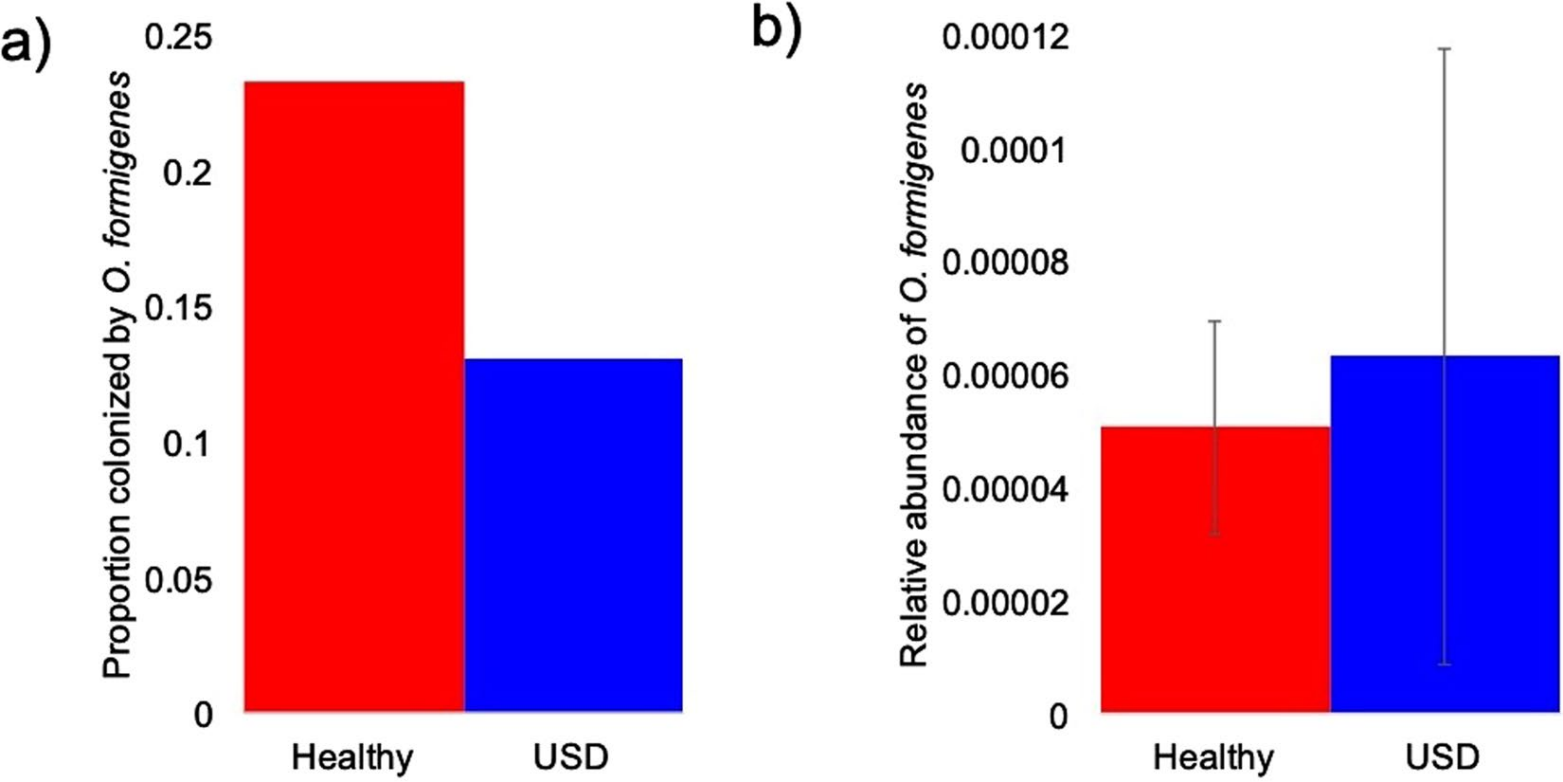
Metrics associated with *Oxalobacter formigenes* between healthy and USD groups. (**a**) Colonization rate of *O. formigenes* between groups. Significance was determined by a relative risk test, followed by a post-hoc Fisher’s exact test (p > 0.05); (**b**) Relative abundance of *O. formigenes*. Significance was determined by a student’s t-test (p > 0.05).

In our dataset, colonization by *O. formigenes* was 23% of healthy individuals and 13% of USD patients, with no significant difference between groups as determined by a relative risk analysis, followed by a post-hoc Fisher’s exact test (Fig. 4a). The relative abundance of *O. formigenes* was also not significantly different by group, as determined by a t-test (Fig. 4b). Furthermore, neither 30-day nor 12-month antibiotic use had a significant correlation to *O. formigenes* colonization.

### Urinary metabolomics by USD-status

**Figure 5:**
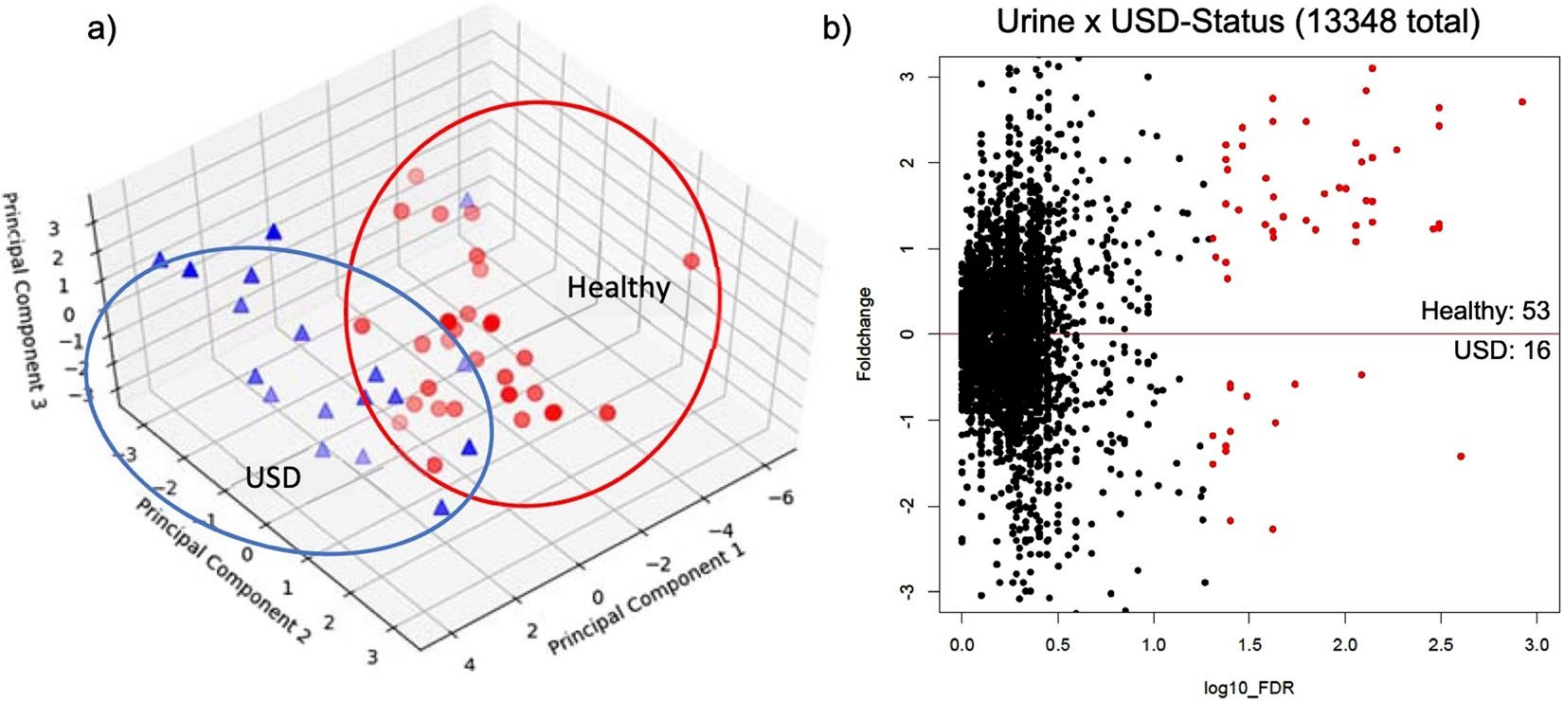
Urinary metabolomic data. (**a**) PCA plot of creatinine-normalized metabolite concentrations by group. (**b**) Metabolites significantly different between healthy and USD groups (red circles). The number of significantly different metabolites are indicated for each group.

Overall, the urinary metabolome clustered by USD-status, based on principal coordinates analysis (PCoA) of the log-transformed, creatinine-normalized, metabolite concentrations (Fig. 5a). When examining the differential concentration of individual metabolites by USD-status, 53 were enriched in the healthy group, with 16 enriched in the USD group, representing a 3.3 fold higher number of metabolites enriched in the healthy cohort compared to the USD cohort (Fig. 5b). Combined, these metabolites made up 0.05% of the total number of metabolites defined in the dataset (Supplementary Table S2).

### Functional microbial networks by specimen-type and USD-status

**Figure 6:**
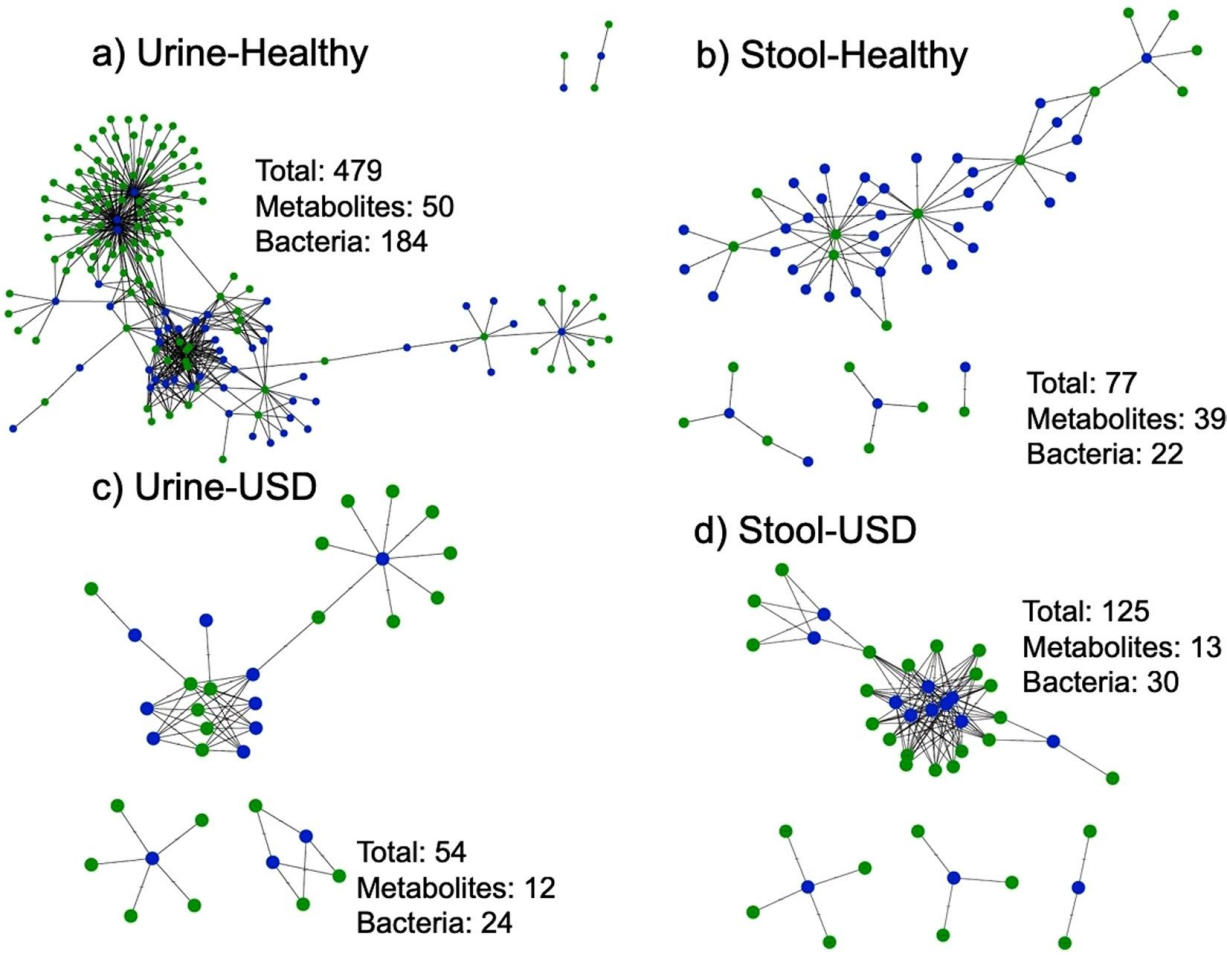
Microbe-Metabolite interaction networks of microbes and metabolites significantly enriched in the healthy or USD groups, for the urine metabolome & both the urine and stool microbiome. Blue = metabolites, green = microbes. Listed are the total number of interactions, number of metabolites involved, and number of bacteria involved. (**a**) Healthy, urine metabolome × urine microbiome; (**b**) Healthy, urine metabolome × stool microbiome; (**c**) USD, urine metabolome × urine microbiome; (**d**) USD, urine metabolome × stool microbiome.

Finally, we wanted to specifically determine what microbe-metabolite interactions most differentiated the healthy population from the USD population. To do so, we integrated the microbiome data with the metabolomic data by conducting pairwise Pearson correlations between the DESeq2-normalized OTU counts that were enriched in either the healthy or USD groups for either the fecal or urinary microbiome and the creatinine-normalized urine metabolite concentrations that were enriched in either the healthy or USD groups. This analysis revealed that what differentiated the healthy cohort from the USD cohort was primarily the loss of *Lactobacillus* from the urinary tract of the healthy population, associated with three currently unknown metabolites (Fig. 6, Table 3).

**Table 3:** List significant microbe-metabolite interactions by group and sample-type.

Metabolite (m/z)	OTU	No. of OTUs/metabolite	Group
Unknown (−387.184893709281)	Lactobacillus	63	Healthy-Urine
Unknown (−406.075648313136)	Lactobacillus	59	Healthy-Urine
Unknown (−299.077321242328)	Lactobacillus	48	Healthy-Urine

Significant positive interactions were determined by pairwise Pearson correlations between OTUs and metabolites significantly enriched in the respective groups (i.e. Healthy-Urine). All FDR-corrected p-values are <0.05, and r > 0.6. Only showing interactions with >10 No. of OTUs/metabolite. Metabolites are listed with their mass:charge (m/z) ratios. Negative values indicate metabolites identified in negative electron spray ionization mode, whereas positive values indicate metabolites identified in positive mode. Group indicates cohort (healthy or USD) and the sample type (urine or stool).

## Discussion

Urolithiasis is an increasingly common disease with diverse pathologies and the factors driving the increase in prevalence are unknown. A recent retrospective analysis found a significant association between oral antibiotic use and USD^57^. Furthermore, several studies have found that patients with USD are colonized by *O. formigenes* at lower rates than healthy individuals^62–69^; that individuals colonized by *O. formigenes* have lower urinary oxalate^62,66–68,70–73^; and that *O. formigenes* is sensitive to several classes of antibiotics^62,69,74,75^. However, a recent meta-analysis of all *Oxalobacter* studies finds that this species is neither necessary nor sufficient by itself to either inhibit USD or lower urinary oxalate^48^. Additionally, recent metagenomic studies find no difference in *O. formigenes* colonization between healthy and USD cohorts^48^, but rather that the USD population harbors a lower diversity of oxalate-degrading bacteria in general, indicating that oxalate-degrading bacteria as a functional group is more important for the prevention of USD than *O. formigenes* alone^47,56^. These findings are in congruence with studies that show oxalate metabolism results from a diverse community of bacteria rather than a single species and provide strong evidence for loss of function dysbiosis as a driver for USD^46,47,50–53^. The above studies all focus on gut bacteria. Outside of struvite stone formation, few studies have focused on the role that urinary tract bacteria may play in the onset of USD^33,34,36,76^. Furthermore, no studies have conducted a direct comparison of the contribution of the gut and urinary tract microbiota to USD and no studies have compared the whole urinary metabolome between healthy and USD patients. Thus, the objective of the current study was to take a multi-site, multi-omics approach to define dysbiosis in a representative population of patients with an active episode of USD, determine which site of microbial activity was most relevant for USD, and which microbe-metabolite interactions may be promoting or inhibiting stone growth.

The results of the current study for the gut microbiota, are largely in congruence with past metagenomic studies (Supplementary Fig. S2), indicative of a consistent form of dysbiosis. Specifically, meta-analysis of these studies in conjunction with those focused on the community of bacteria associated with oxalate metabolism finds that the taxa reduced in the gut microbiota of USD patients largely overlaps with the oxalate-degrading microbial network (Supplementary Fig. S2, Table S3). Furthermore, in congruence with metagenomic studies but in contrast to many studies that determined *O. formigenes* colonization through culture-based or PCR-based methods, we did not find a difference in *O. formigenes* colonization between our healthy and USD cohorts, nor did we find a negative correlation between *O. formigenes* colonization and antibiotic use (Fig. 4). It is not currently known why there is this difference in metagenomic and culture- or PCR-based methods of detection.

The current study is the first metagenomics study to compare the urinary tract microbiome between USD and healthy populations. Several lines of evidence in this study point to the urinary tract microbiome as a greater contributor to the onset of USD than the gut microbiota. First, statistical analysis of the microbiota composition reveals that the urinary tract microbiota, but not the gut microbiota, was significantly different by USD-status (Fig. 3a,b, Table 2). These results are corroborated by differential abundance analysis that showed a greater proportion of OTUs from the urinary tract were significantly different between the USD and healthy cohorts compared to the gut (Fig. 3c,d, Supplementary Table S1). Secondly, the urinary tract microbiota composition, but not the gut microbiota was also significantly different based on antibiotic use, family history of USD, and sex (Table 2). These factors have all been associated with USD in the past^57,60,77^. Third, the overlap between the taxonomic profile of the kidney stone microbiota and that of the urine from USD patients was much greater than the taxonomic profile of the urine between the healthy and USD cohorts (Supplementary Fig. S1). This result raises the possibility that bacteria in the urinary tract of people at risk for USD may play a direct role in stone formation. Finally, by integrating the microbiome data with the urinary metabolome data, we find that what differentiates the USD and healthy cohorts the most is the microbe-metabolite networks of the urinary tract microbiome and urinary metabolome. The metabolome is the end result of human and microbe metabolic processes^78,79^. Furthermore, the urinary metabolome specifically is a known risk factor for USD that is often targeted in metabolic analyses^80^. Thus, integration of microbiome and metabolome data allows us to hone in on the most important microbe-metabolite interactions for USD. Specifically here, the association between *Lactobacillus* bacteria and three currently unknown metabolites was the most differentiating factor from the integrated datasets (Table 3, Supplementary Table S1). However, bacteria from the *Enterobacteriaceae* family had a strong association with the urinary tract of the USD cohort (Supplementary Table S1). Interestingly, while many clinics often perform bacterial analysis on urine and kidney stones, our results show a strong bias of culture results compared to when DNA is extracted directly from stone or urine samples (Fig. 2, Supplementary Figs 1 and 3). Specifically, there was an apparent culture bias against the *Firmicutes, Actinobacteria*, and rare phyla (Supplementary Fig. S1). Importantly, the *Lactobacillus*, which were found to be greatly reduced in the USD population, is part of the *Firmicutes* phylum. Thus, culture-based approaches to microbial profiling in the urine and kidney stones may overlook important features of the urinary tract microbiome in association with USD, as has been noted previously^76^.

Evidence is increasingly mounting as to the health-protective and disease promoting effects of urinary tract *Lactobacillus* and *Enterobacteriaceae*, respectively. Specifically, the *Lactobacillus* are typically found in the urinary tract of healthy individuals^39^ and a recent clinical trial found that urinary tract *Lactobacillus* helped to prevent UTI’s^38^. In contrast, most bacteria associated with UTI’s and struvite stones come from the *Enterobacteriaceae* family^8,31–36^. While we excluded patients with struvite stones for the current study, we still found a strong association with *Enterobacteriaceae* in the urine of the USD cohort, indicating that bacteria from this family may generally promote stone growth in the urinary tract. These results are in congruence with a recent study that found a strong association between the *Enterobacteriaceae* in the urine and kidney stones of calcium oxalate stone formers and that the presence of *Enterobacteriaceae* and calcium oxalate together had a synergistic effect on renal calcium oxalate deposition^76^.

The results of the current study in conjunction with previous reports allows us to devise a specific, testable model for the contribution of the microbiome to the onset of USD. The model suggested by the current study is based on a combination dysbiosis model in which we propose that antibiotics reduce colonization of oxalate-degrading bacteria or other functional groups in the gut, as shown in recent animal studies^61^, and would lead to higher levels of serum and urinary oxalate or other stone-promoting metabolites. In the urinary tract, we propose that antibiotics lead to a loss of health-protective *Lactobacillus* and a subsequent re-colonization by stone-promoting *Enterobacteriaceae*. The persistent loss and colonization of urinary tract *Lactobacillus* and *Enterobacteriaceae*, respectively, after a course of antibiotics has been demonstrated before^81^. It is currently unknown how the *Lactobacillus* may inhibit stone growth. However, our multi-omics integration strongly points to three unknown metabolites. Future work will elucidate the structure of these metabolites, based on the mass:charge ratio reported (Table 3) and their effect on stone growth. It is likewise unknown how *Enterobacteriaceae* promote stone growth. However, the study by Barr-Beare *et al*. (2015) point to a direct role for these bacteria in the aggregation of metabolites into the stone matrix.

Urinary stone disease represents diverse pathologies, likely with equally diverse causal mechanisms that lead to stone formation. In the current study, we recruited patients with different stone types that include calcium oxalate, calcium phosphate, uric acid stones, and some composite stones, specifically to determine if there was an underlying association between dysbiosis with the microbiome and the onset of USD. While our results strongly suggest a common dysbiotic link between the microbiome and different pathologies of USD, it is likely that the specific groups of bacteria lost/gained in the gut or urinary tract contributes to the type of stone that manifests in the patient. However, more work is needed to elucidate these different mechanistic pathways.

To conclude, the current study provides the most direct and proximate link between antibiotic use, the microbiome, and USD. Results of the study provide strong evidence for a combination of loss and gain of function dysbiosis centered on oxalate metabolism in the gut and *Lactobacillus/Enterobacteriaceae* in the urinary tract. Prospective, longitudinal clinical trials are now needed to test and confirm this hypothesized model.

## Methods

### Recruitment of participants

Patients who had an active episode of USD were given the option to participate in the current study. Control subjects without a history of USD were recruited by the clinical research unit (CRU) at Cleveland Clinic. All subjects were required to fill out a questionnaire detailing information associated with health, diet, and use of medications (Supplemental File 1). Exclusion criteria included prior personal history of USD (healthy cohort only), chronic gastrointestinal issues, and age (<18 years old). We did not exclude patients on the basis of diet, age (>18 years old), or medications (antibiotics or otherwise) in order to test hypotheses associated with factors that impact the microbiome in ways that could facilitate the onset of USD. The prospective clinical cohort were representative of the typical USD population relative to stone composition, age, and presence of co-morbidities (Table 1).

### Sample collection and processing

Each subject was asked to provide a stool sample and a voided urine sample. Stool samples were self-collected by study subjects using a provided rectal swab containing modified Cary-Blair medium. Voided, clean-catch mid-stream urine was collected from all subjects, either in clinic or in the preoperative area prior to the stone procedure and pre- or perioperative antibiotics. From the urine sample in culture & sensitivity preservative (BD Scientific), 200 ul was used for cell culture and the remainder was used for DNA extraction. Urine, stool, and stone samples were stored in preservative at 4 °C prior to processing within 24 hours of collection.

Stone samples were collected after surgical procedure for removal (uteroscopy or percutaneuous nephrolithotomy), with a portion of the sample sent for clinical analysis of composition. Remaining stone samples were rinsed with sterile PBS to remove potential host bacteria contamination, flash frozen in liquid nitrogen and pulverized with a sterile mortar and pestle. Half of the pulverized stone was suspended in 15% glycerol and stored at −80 °C before culturing and the remainder of the pulverized stone was used for DNA extraction.

For cultures, 100 uL of urine or stone powder submerged in glycerol was inoculated to Columbia Blood Agar (Edge Biologicals, Memphis, TN) and MacConkey’s agar (Oxoid Agar Biological, ThermoFisher Scientific) plates. Bacteria were incubated aerobically at 37 °C for up to 5 days and were monitored daily for growth. Colonies were picked using a flame sterilized loop and suspended in 1 mL PBS. Culture conditions were designed to mimic culture conditions in typical clinical practices^82^.

Urine, urine culture, and stone culture DNA was isolated using the Urine DNA Isolation Kit for Exfoliated Cells or Bacteria (Norgen, Thorold, ON, Canada). Prior to extraction, the urine sample was centrifuged 15,000 g for at least five minutes and the culture samples in PBS were centrifuged at 14000 g for three minutes. Pellets were re-suspended and mixed with 600 ul lysis buffer B, 12 ul lysozyme stock, 10 ul Proteinase K, and 20 ul mutanolysin. The mixture was incubated at 37 °C for 60 minutes, with vortexing every 15 minutes. The remainder of the protocol was followed according to the manufacturer’s instructions.

To ensure consistent extraction of DNA from kidney stones, a modified protocol from the Qiagen DNeasy Blood & Tissue Kit was developed (QIAGEN GmbH, Hilden, Germany). Specifically, buffer ATL was added to cover a pulverized stone sample and incubated with lysozyme, mutanolysin, and protease K at 37 °C for 1 hour, vortexing every 15 minutes. Subsequent processing was performed according to the manufacturer’s protocol.

Approximately 0.25–1 g of fecal samples were recovered from fecal swabs after centrifugation and collection of the pellet. QIAamp PowerFecal DNA Kit (QIAGEN GmbH, Hilden, Germany) was used for DNA extraction. For all DNA extractions, negative controls that included sterile water first placed into collection vessels and all extraction reagents were performed in conjunction with every round of extractions. Subsequently, all extractions were verified with gel electrophoresis and concentrations were quantified with a Nanodrop Spectrophotometer (Thermo Scientific). Only samples that exhibited the presence of a band on gel electrophoresis and had a DNA concentration >15 ng/ul were submitted for sequencing. No negative controls from any preparation had any quantifiable DNA. While all stool samples had quantifiable DNA, only 64 of the 67 urine samples and 10 of the 15 stone samples had detectable DNA that was used in downstream sequencing.

Urine samples were prepared for untargeted metabolomics by diluting each sample 1:4 in a 50% acetonitrile solution containing two internal standards, 30 uM 4-nitrobenzoic acid (Acros Organics, Fair Lawn, NJ, USA), and 2 uM debrisoquine (Santa Cruz Biotechnology, Dallas, TX, USA)^83^. Samples were centrifuged at 18,000 rcf for 5 minutes to precipitate proteins, and the supernatant was recovered and stored at −80 °C prior to analysis.

### DNA sequencing and analysis

Extracted DNA from feces, urine, and kidney stones was sent to Argonne National Laboratory (Chicago, IL) for sequencing of the V4 region of the 16S rRNA gene on the Illumina MiSeq platform after amplification with the 515F and 806R primers^84^. Barcodes with 12 base pairs were added to the amplified region and samples multiplexed on a single lane for 150 base pair, paired-end sequencing^84^.

Raw sequencing data were demultiplexed and quality-controlled with default parameters in QIIME^85^. Operational taxonomic units (OTUs) were assigned with open reference assignment, with 97% homology compared to a reference database composed of the Greengenes dataset and from previous datasets of *de novo* assigned OTUs, to permit direct comparison across studies^50,51,86^. All OTUs that did not exhibit a match from the reference database were classified *de novo*. Sequences associated with chloroplasts, mitochondria, chimeras, or that had <10 representations across the dataset for each sample type were removed prior to downstream analyses, as previously described^50,51^.

Data were normalized with a negative binomial Wald test through the DESeq. 2 algorithm prior to diversity analyses^87,88^. For α−diversity, Margalef’s species richness, equitability, Shannon’s Index, and Phylogenetic diversity were quantified. Statistical analysis of α-diversity was calculated through paired t-tests in R statistical software^89^. For β−diversity, both weighted and unweighted UniFrac distances were calculated and statistical analysis was conducted through a Permanova analysis (Adonis), with 999 permutations^90^. Differential abundance analysis was conducted through a Wald test, which determines significance through the log_2_ fold change of normalized OTU abundance between groups, divided by standard error. The p-values were then adjusted to account for false discoveries (FDR)^87^. The network of bacteria that co-occur with *Oxalobacter formigenes* was performed as previously described^51,91^. Briefly, the relative abundance of OTUs was correlated to the relative abundance of *O. formigenes*, using FDR-corrected Pearson correlations.

### Untargeted metabolomics and analysis

Urine from 18 USD subjects and 31 control subjects was available for metabolomic analysis. After the samples were prepared as above, they were submitted for processing via liquid chromatography/tandem mass spectroscopy (LC-MS-MS). External standards were added to the samples prior to injection onto the Vanquish UHPLC system coupled to a Q Exactive HF hybrid quadrupole-orbitrap mass spectrometer (Thermo Scientific, Waltham, MA). The mass spectrometer was operated in positive and negative electrospray ionization modes over a mass range of 50–750 Da. The XCMS software package was used to de-convolute the raw data^92^. The detected ions were normalized to total creatinine and further analyzed using Metabolyzer software^93^. Concentrations were quantified with comparison to the two internal standards added at a known concentration. All metabolites were defined by ionization, molecular mass, and retention time (m/z) in the UHPLC-MS-MS system. Metabolites were given putative identification by comparison with metabolites in the KEGG, HMDB, LIPIDMAPS, and BioCyc databases^94–97^.

### Integration of 16S rRNA and metabolomic data

To integrate the 16S rRNA and metabolomic data, we used OTUs significantly enriched in the (1) urine of the healthy group; (2) urine of USD group; (3) stool of the healthy group; or (4) stool of the USD group. Normalized counts of these OTUs were integrated with the significantly different metabolites from the (1) urine of the healthy group; or (2) urine of the USD group. Correlation networks were calculated by conducting all pairwise microbe-metabolite Pearson correlations. Only correlations > 0.6 and with an FDR-corrected p-value < 0.05 were used in downstream analyses. Correlation networks were generated from: (1) The urine microbiome and urine metabolome of the healthy group; (2) The urine microbiome and urine metabolome of the USD group; (3) The fecal microbiome and urine metabolome of the healthy group; and (4) The fecal microbiome and urine metabolome of the USD group. Resulting microbe-metabolite networks were visualized in Cytoscape.

### Ethics approval and consent to participate

All study procedures were approved by the Institutional Review Board of Cleveland Clinic (IRB# 16–643). All research was performed in accordance with relevant guidelines and regulations. Subjects provided written informed consent prior to participating in the study.

## Supplementary information

Table S1

Table S2

Table S3

Patient questionairre

## Data Availability

Sequence reads are available at the Sequence Read Archive under Accession # SRP140641.
