# Supplementary material for: Defining Dysbiosis in Patients with Urolithiasis
Source: Sci Rep. 2019 Apr 1;9:5425. doi: 10.1038/s41598-019-41977-6 (PMC6443657; doi:10.1038/s41598-019-41977-6)

Defining Dysbiosis in Patients with Urolithiasis

Anna Zampini, M.D.*[,1], Andrew H. Nguyen, M.D.*[,1], Emily Rose, B.S.[2], Manoj Monga, M.D.[1], Aaron W. Miller, Ph.D.[1,3,#]

*Co-First authors

[1]Glickman Urological and Kidney Institute, Cleveland Clinic, Cleveland, OH, USA

[2]Lerner College of Medicine, Cleveland Clinic, Cleveland, OH, USA

[3]Department of Inflammation and Immunity, Lerner Research Institute, Cleveland Clinic, Cleveland, OH, USA

#*Corresponding author:*

Miller A.W.,

Lerner Research Institute,

Glickman Urological and Kidney Institute,

Cleveland Clinic

millera25@ccf.org

Phone: 1 216 444 1721

Fax: 1 216 444 9329

Running head: Dysbiosis in Urolithiasis

Key words: urolithiasis, metagenomics, metabolomics, dysbiosis

Patient Name ____

Patient Questionnaire for the Microbial Network Indicators of Urinary Stone Disease (MiNIU) and Longitudinal Cohort for Microbiome and Urinary Stone Disease
The MiNIU is intended to collect a snapshot of the microbiome in eligible patients and their family members. Your answers will be anonymous.

Please answer the following questions as completely as possible:

What is your age (in years)? ____

What is your biologic gender? (male or female)
____

What is your height (feet and/or inches)? ____

What is your weight (lbs)? ____

Please complete the following questions by circling the best answer:

1. Have you ever had a kidney stone?
(Please circle Yes or No) Yes No

2. Does anyone in your family have kidney stones?
(Please circle Yes or No) Yes No

If you answered "Yes" to the above question, which family member has kidney stones?

____

3. Do you have, or have you ever had a chronic gastrointestinal illness?
(Please circle Yes or No) Yes No

4. Are you taking a daily medication for a gastrointestinal illness or condition?
(Please circle Yes or No) Yes No

5. Have you taken daily medication for a gastrointestinal illness or condition in the past?
(Please circle Yes or No) Yes No

Patient Name ____

Patient Questionnaire for the Microbial Network Indicators of Urinary Stone Disease (MiNIU) and Longitudinal Cohort for Microbiome and Urinary Stone Disease
The MiNIU is intended to collect a snapshot of the microbiome in eligible patients and their family members. Your answers will be anonymous.

If you answered "Yes" to the previous question, what medication(s) did you take?

____

6. Have you used or taken any antacids in the past 30 days?
(Please circle Yes or No) Yes No

7. Have you used or taken a probiotic in the past 30 days?
(Please circle Yes or No) Yes No

8. Have you used or taken any vitamins, minerals, herbals supplements or other dietary supplements in the past 30 days?
(Please circle your answer) Yes No I don't know

If you answered "Yes" to the previous question, what vitamins, minerals, herbals supplements or other dietary supplements did you take?

____

9. Do you take stool softeners or laxatives (either supplements or medications)?
(Please circle Yes or No) Yes No

If you answered "Yes" to the previous question, what stool supplements or medications do you take?
____

10. Have you used or taken an antibiotic in the last year (12 months)?
(Please circle your answer) Yes No I don't know

11. Have you used or taken an antibiotic in the past month (30 days)?
(Please circle your answer) Yes No I don't know

12. Have you been diagnosed with gout?
(Please circle Yes or No) Yes No

Patient Name ____

Patient Questionnaire for the Microbial Network Indicators of Urinary Stone Disease (MiNIU) and Longitudinal Cohort for Microbiome and Urinary Stone Disease
The MiNIU is intended to collect a snapshot of the microbiome in eligible patients and their family members. Your answers will be anonymous.

13. Have you been diagnosed with diabetes?
(Please circle Yes or No) Yes No

14. Do you have high blood pressure?
(Please circle Yes or No) Yes No

15. Will you be willing and able to provide urine and stool samples for bacterial analysis?
(These samples may be collected at home and you will be provided with a specific collection kit.)
(Please circle Yes or No)
Yes No

16. How would you describe your diet?
(Please circle one option)

Vegan Vegetarian Omnivore (Meat and Vegetables)

Low Carbohydrate Diet Mediterranean Diet Paleolithic Diet

Pescatarian (Fish)

17. How much water do you drink each day?
(Please circle your answer in number of glasses)

1 2 3 4 5 6 7 8 9 10 11 12 > 12 glasses

18. Do you eat sweets, candies, pastries or desserts daily?
(Please circle your answer) Yes No

19. On average how many times per week do you eat meat (of any kind)?
(Please circle the most appropriate answer)

0 1-2 3-4 5-6 7-8 8-9 10-14 14 times per week

Patient Name ____

Patient Questionnaire for the Microbial Network Indicators of Urinary Stone Disease (MiNIU) and Longitudinal Cohort for Microbiome and Urinary Stone Disease
The MiNIU is intended to collect a snapshot of the microbiome in eligible patients and their family members. Your answers will be anonymous.

20. On average how many servings of fruit do you eat per day ?
0 1-2 3-4 5-6 7-8 8-9 >10 times per day

21. On average how many servings of vegetables or salad do you eat per day
0 1-2 3-4 5-6 7-8 8-9 >10 times per day

22. How many servings of white bread, rice or pasta do you eat per day?
0 1-2 3-4 5-6 7-8 8-9 >10 times per day

THANK YOU FOR COMPLETING THIS SURVEY

Figure S1. Phylum-level profile of the microbiome by USD-status. A) Phylum profile by specimen-type, of samples that only underwent molecular analysis. B) Phylum profile comparing molecular only vs. samples that were cultured prior to molecular analysis.

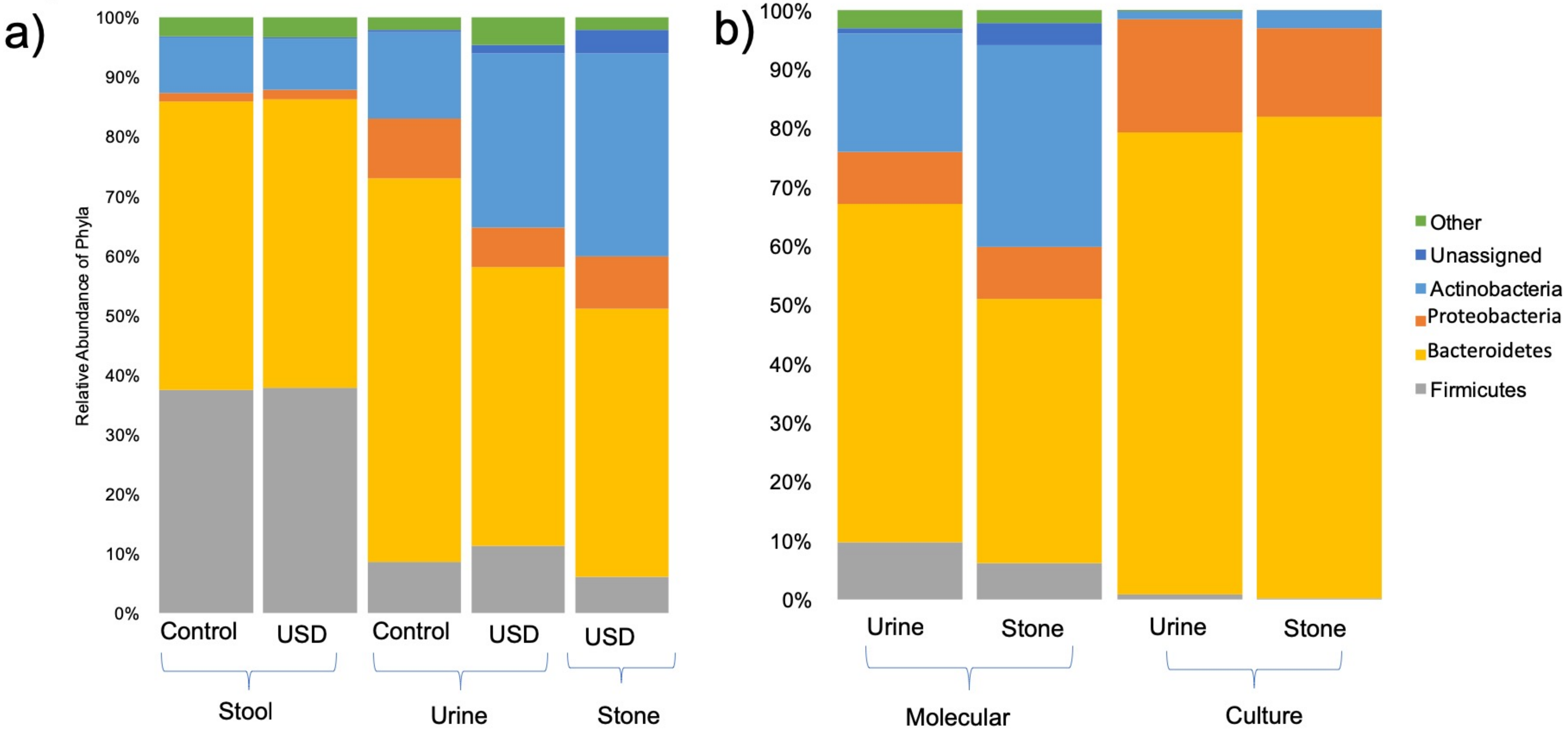

Figure S2. Meta-analysis of all studies that have examined the whole gut microbiota in terms of the OTUs associated with the oxalate-degrading microbial network (ODMN) or enriched/depleted in the USD groups, summarized to genus-level taxonomy. Genera are ordered from those that are enriched the most often (top) to least often (bottom). Primary data sources were independently analyzed if available. Red box indicates studies in humans. *Primary data not available.

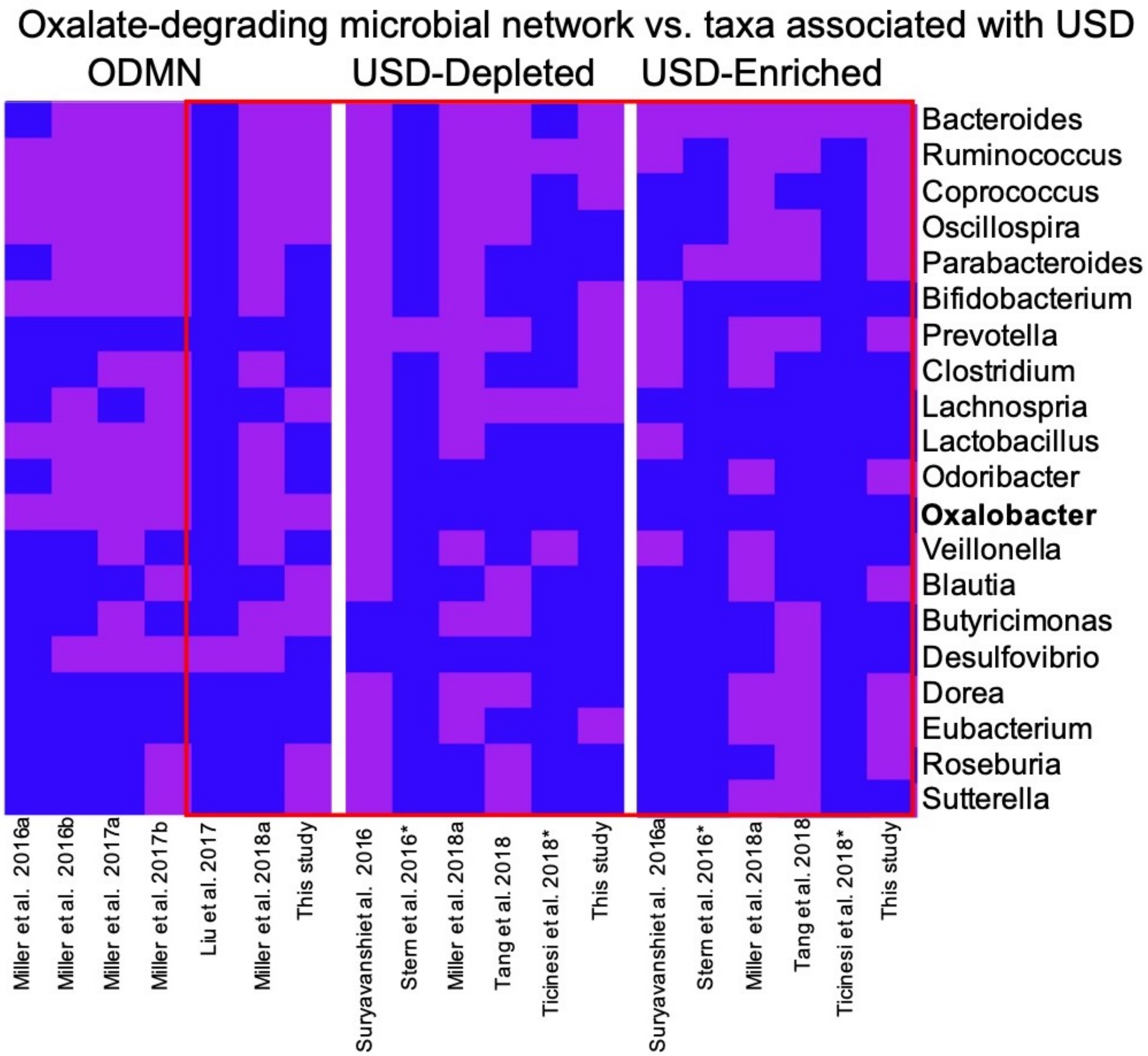

Figure S3. Phylogenetic diversity comparing techniques to examine the microbiota in urine and stone. Significant p-values are listed next to groups that exhibited a difference by technique. Significance was determined by a student's t-test. A) Species richness; B) Evenness; C) Shannon's index; D) Phylogenetic diversity.

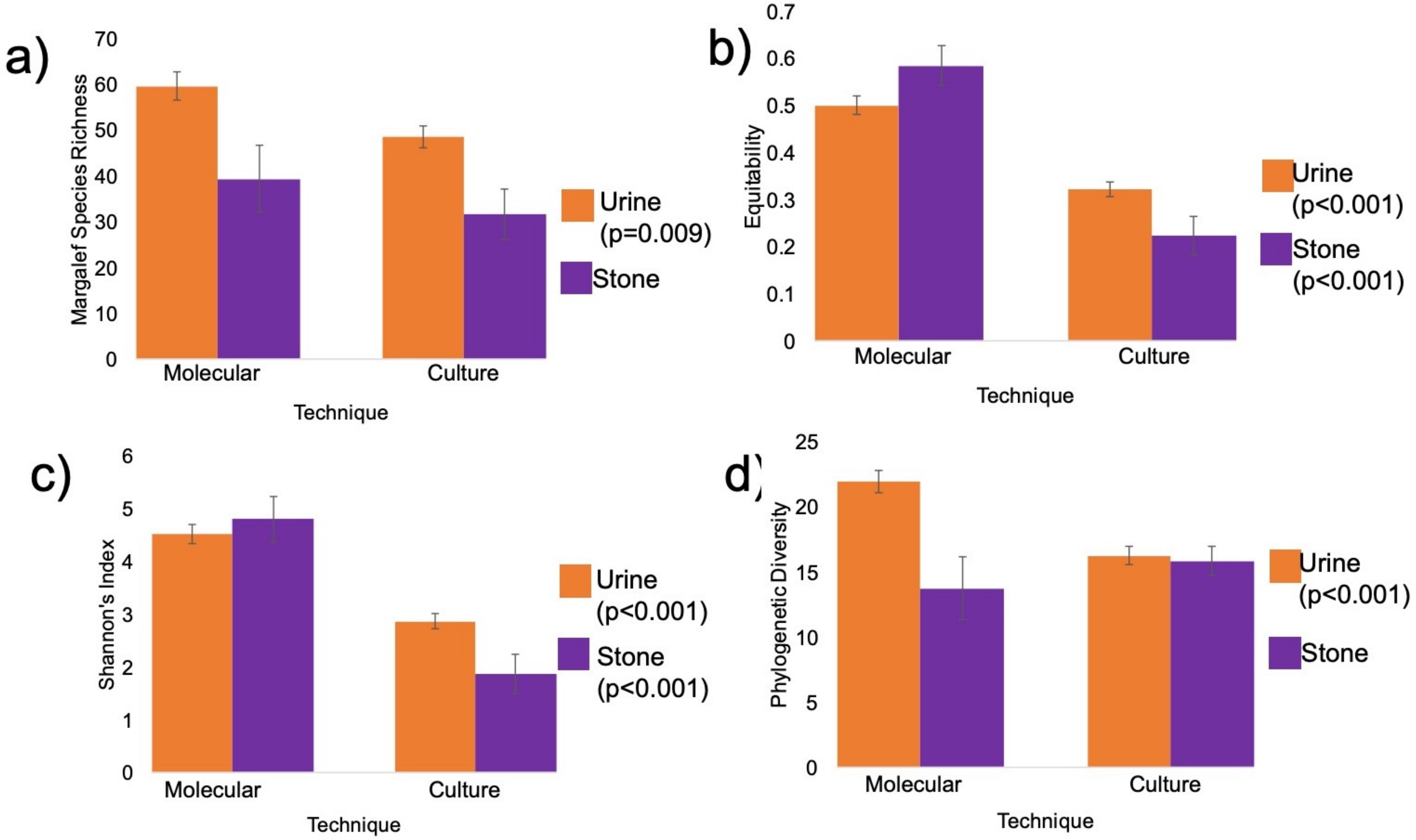

Supplement: Supplementary file 4 — Patient questionairre [file 41598_2019_41977_MOESM4_ESM.pdf]
